# Effects of Walnut Consumption on Blood Lipid Profile and Apolipoproteins in Adults: A GRADE‐Assessed Systematic Review and Dose–Response Meta‐Analysis of 49 Randomized Controlled Trials

**DOI:** 10.1002/fsn3.71526

**Published:** 2026-02-10

**Authors:** Ghazal Mashayekhi, Damoon Ashtary‐Larky, Mehdi Karimi, Omid Asbaghi, Arvin Porkar Rezaeyeh, Zahra Shouhani, Ali Hosseini, Moslem Naderian

**Affiliations:** ^1^ Department of Nutrition Kerman University of Medical Sciences Kerman Iran; ^2^ Nutrition and Metabolic Diseases Research Center Ahvaz Jundishapur University of Medical Sciences Ahvaz Iran; ^3^ Faculty of Medicine Bogomolets National Medical University Kyiv Ukraine; ^4^ Cancer Research Center Shahid Beheshti University of Medical Sciences Tehran Iran; ^5^ School of Medicine Iran University of Medical Sciences Tehran Iran; ^6^ School of Medicine Ahvaz Jundishapur University of Medical Science Ahvaz Iran; ^7^ Department of Pharmacognosy, School of Pharmacy Shiraz University of Medical Sciences Shiraz Iran; ^8^ Medicinal Plants Research Center Yasuj University of Medical Sciences Yasuj Iran

**Keywords:** apolipoprotein, cholesterol, *Juglans regia*, lipid profile, meta‐analysis, triglycerides, walnut

## Abstract

Walnuts ( *Juglans regia* L.) are rich in polyunsaturated fatty acids (PUFAs), fiber, and bioactive compounds that may positively affect lipid metabolism. Although several clinical trials have examined their impact on the lipid profile, results have been inconsistent, and evidence regarding their effects on apolipoproteins is limited. This meta‐analysis aims to systematically assess the impact of walnut consumption on blood lipid profile and apolipoproteins in adults. A comprehensive search of major databases was conducted up to August 2025 to find relevant randomized controlled trials (RCTs) evaluating the effects of walnut intake on triglycerides (TG), total cholesterol (TC), low‐ and high‐density lipoprotein cholesterol (LDL‐C and HDL‐C), apolipoproteins A1 and B (Apo‐A1 and Apo‐B). Pooled effect sizes were estimated using a random‐effects model. The pooled analysis of 49 RCTs (57 effect sizes; 4611 participants) showed that Walnut consumption significantly reduced and TG (WMD: −6.24 mg/dL, 95% CI: [−9.49, −2.99], *p* < 0.001), TC (WMD: −6.39 mg/dL, 95% CI: [−8.54, −4.23], *p* < 0.001), and LDL‐C (WMD: −5.68 mg/dL; 95% CI: [−7,89, −3.47], *p* < 0.001), while no significant effect was observed on HDL‐C (WMD: 0.71 mg/dL, 95% CI: [−0.24, 1.68], *p* = 0.145), Apo‐A1 (WMD: 0.0, 95% CI: [−0.01, 0.00], *p* = 0.573), or Apo‐B (WMD: 0.1, 95% CI: [−0.02, 0.05], *p* = 0.462). However, subgroup analyses indicated significant reductions in Apo‐B at higher walnut doses (> 50 g/day) and increases in HDL‐C in the low‐dose intervention (< 50 g/day). In conclusion, walnut supplementation improves the lipid profile by significantly lowering TC, LDL‐C, and TG levels. However, effects on HDL‐C and apolipoproteins remain inconclusive. These findings support the incorporation of walnuts as part of a heart‐healthy dietary pattern.

## Graphical abstract

Background: Walnuts (*Juglans regia L.*) are rich in PUFAs, fiber, and bioactive compounds with potential lipid‐lowering effects, but evidence—especially for apolipoproteins—remains inconsistent. Methods: Systematic review and meta‐analysis of 49 RCTs up to August 2025 assessing walnut intake and blood lipids (TG, TC, LDL‐C, HDL‐C) and apolipoproteins (Apo‐A1, Apo‐B). Results: Walnut consumption significantly reduced TG, TC, and LDL‐C, with no overall effect on HDL‐C, Apo‐A1, or Apo‐B. Subgroup analyses showed that Apo‐B reduction was observed at doses > 50 g/day and that HDL‐C increased at doses < 50 g/day. Conclusion: Walnuts improve the lipid profile, supporting their inclusion in a heart‐healthy diet, whereas effects on apolipoproteins remain dose‐dependent and inconclusive.

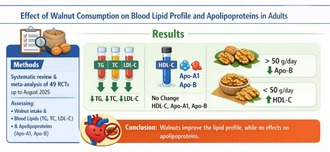

**Abbreviations**
Apo‐A1: apolipoproteins A1
Apo‐B: apolipoproteins B
HDL‐C: high‐density lipoprotein cholesterol
LDL‐C: low‐density lipoprotein cholesterol
RCT: randomized controlled trials
TC: total cholesterol
TG: triglycerides
VLDL‐C: very low‐density lipoprotein cholesterol

## 1 Introduction

Cardiovascular disease remains the foremost cause of morbidity and mortality worldwide (Chong et al. 2025). One of its principal risk factors is dyslipidemia, a metabolic disorder defined by elevated plasma levels of triglycerides (TG), total cholesterol (TC), low‐density lipoprotein cholesterol (LDL‐C), and apolipoprotein B (Apo‐B), alongside reduced concentrations of high‐density lipoprotein cholesterol (HDL‐C) and apolipoprotein A1 (Apo‐A1) (Arvanitis and Lowenstein 2023). The development of dyslipidemia reflects a complex interplay between genetic susceptibility and environmental influences, ultimately driving atherosclerotic progression and cardiovascular complications (Abe et al. 2015). Globally, more than one‐quarter of adults are affected by dyslipidemia, which often coexists with metabolic syndrome and atherosclerosis (Ballena‐Caicedo et al. 2025; Grundy 2006).

In recent years, nutritional strategies have emerged as promising non‐pharmacological approaches for optimizing lipid profiles and promoting cardiovascular health (Karimi, Javadi, Hamzavi, et al. 2025; Karimi et al. 2024; Karimi, Bahreini, et al. 2025). Among these, nut consumption, particularly walnuts (*Juglans regia L*.), has attracted considerable attention due to their unique nutrient composition. Walnuts are rich in polyunsaturated fatty acids (PUFAs), especially alpha‐linolenic acid (ALA), and also provide plant‐based protein, dietary fiber, polyphenols, and a variety of bioactive compounds with the potential to mitigate inflammation, oxidative stress, and lipid dysregulation (Martínez et al. 2010; Fan et al. 2023; Fizeșan et al. 2021). Despite this strong nutritional rationale, findings from randomized controlled trials (RCTs) evaluating the lipid‐modulating effects of walnut consumption have been inconsistent. For example, Tapsell et al. reported no significant changes in lipid parameters following daily walnut intake in patients with type 2 diabetes (Tapsell et al. 2017). Likewise, Hwang et al. (2019) found that 16 weeks of walnut supplementation increased HDL‐C levels but did not affect other lipid markers in individuals with metabolic syndrome. In contrast, Bashan et al. demonstrated that three months of walnut consumption significantly reduced TC, LDL‐C, very low‐density lipoprotein cholesterol (VLDL‐C), and TG, while increasing HDL‐C in adults (Bashan and Bakman 2018).

Previous meta‐analyses have sought to consolidate evidence on the lipid‐modulating effects of walnuts; however, most have been constrained by a limited number of included trials and have overlooked key lipoprotein‐related biomarkers. For instance, Guasch‐Ferré et al. (2018), in a systematic review of 26 clinical trials, reported that walnut consumption was associated with reductions in TC, LDL‐C, and TG, but showed no significant effect on HDL‐C. Similarly, Alshahrani et al. (2022) in a more recent meta‐analysis of 13 studies confirmed significant decreases in TC, LDL‐C, and TG, without meaningful changes in HDL‐C.

Despite these insights, previous analyses have not evaluated key apolipoproteins such as Apo‐A1 and Apo‐B, which are stronger indicators of lipoprotein particle number and cardiovascular risk (Andrikoula and McDowell 2008). This gap, along with the availability of newer randomized clinical trials, underscores the need for an updated, more comprehensive synthesis. Therefore, the present systematic review and meta‐analysis aim to (Chong et al. 2025) evaluate the effects of walnut consumption on apolipoproteins (Apo‐A1 and Apo‐B), and (Arvanitis and Lowenstein 2023) re‐assess its impact on conventional serum lipid markers, including TC, LDL‐C, HDL‐C, and TG to provide a clearer and more integrated understanding of the potential role of walnuts in lipid regulation and cardiovascular risk reduction.

## 2 Methods

This systematic review and meta‐analysis were conducted in accordance with the Preferred Reporting Items for Systematic Reviews and Meta‐Analyses (PRISMA) guidelines (Moher et al. 2009).

### 2.1 Search Strategy

A comprehensive search was conducted across three major electronic databases, PubMed, Scopus, and Web of Science, from inception through August 2025. The search strategy utilized both Medical Subject Headings (MeSH) and free‐text terms as follows: (Walnut[Title/Abstract] OR Walnuts[Title/Abstract]) AND (trials[Title/Abstract] OR “clinical trial”[Title/Abstract] OR “controlled trial”[Title/Abstract] OR randomized[Title/Abstract] OR randomly[Title/Abstract] OR random[Title/Abstract] OR Intervention[Title/Abstract] OR placebo[Title/Abstract] OR blinded[Title/Abstract] OR parallel[Title/Abstract] OR “Cross‐Over”[Title/Abstract]). No restrictions were applied regarding the date or language of publication. Additionally, the reference lists of all relevant articles were manually screened to identify any additional eligible studies. All retrieved citations were imported into EndNote, a reference management software, for screening. Duplicates and unpublished records were subsequently excluded.

### 2.2 Inclusion Criteria

Studies were considered eligible if they met the following criteria: (Chong et al. 2025) RCT design, (Arvanitis and Lowenstein 2023) participants aged 18 years or older, (Abe et al. 2015) interventions involving walnut consumption, and (Ballena‐Caicedo et al. 2025) assessment of lipid profile parameters and/or apolipoproteins in both intervention and control groups. In cases where multiple publications reported findings from the same dataset, the most comprehensive or recent version was included. Trials that included multiple intervention arms were treated as separate comparisons if relevant.

### 2.3 Exclusion Criteria

Studies were excluded if they: (Chong et al. 2025) employed observational designs such as cohort, case–control, or cross‐sectional studies; (Arvanitis and Lowenstein 2023) were review articles; (Abe et al. 2015) were ecological studies; (Ballena‐Caicedo et al. 2025) lacked a control group or were non‐randomized; or (Grundy 2006) involved pediatric or adolescent populations.

### 2.4 Data Extraction

Data extraction was independently conducted by two reviewers (G.M. and Z.S.). The following information was obtained from each included trial: first author's name, year of publication, country of study, study design, sample size per group, participant characteristics (e.g., mean age, sex distribution, body mass index), intervention details (e.g., walnut dose, duration), outcome measures (e.g., mean changes and standard deviations in TG, TC, LDL‐C, HDL‐C, Apo‐A1, and Apo‐B), and confounding factors adjusted for in the analyses. When necessary, units were standardized to ensure consistency across studies.

To address multi‐arm trials and avoid double‐counting, when an RCT included more than one relevant intervention arm, or when multiple effect sizes could be derived from the same dataset (e.g., different walnut doses or different populations), each eligible comparison was treated as an independent effect size. This approach allowed multi‐arm RCTs to contribute multiple comparisons to the pooled analysis; however, the number of participants in shared control groups was not duplicated across any single analysis. Consequently, the total number of effect sizes exceeded the number of included studies.

### 2.5 Quality Assessment

The methodological quality of the included RCTs was independently evaluated by two reviewers using the Cochrane Risk of Bias tool (Sterne et al. 2019), which assesses bias across domains such as randomization, allocation concealment, blinding, incomplete outcome data, selective reporting, and other potential sources of bias. Discrepancies between reviewers were resolved through discussion with the corresponding author.

### 2.6 Statistical Analysis

For this meta‐analysis, weighted mean differences (WMDs) and their corresponding standard deviations (SDs) for TG, TC, LDL‐C, HDL‐C, Apo‐A1, and Apo‐B were extracted from the intervention and control groups of each included study. When the mean change was not explicitly reported, it was calculated as the difference between post‐intervention and baseline values. The SD of the change was derived using the following equation (Borenstein et al. 2011):

$$\mathrm{SD}\;\text{change}=\sqrt{\left[{\left(\mathrm{SD}\;\text{baseline}\right)}^{2}+{\left(\mathrm{SD}\;\text{final}\right)}^{2}\right]-\left(2R\times \mathrm{SD}\;\text{baseline}\times \mathrm{SD}\right)}$$

where *R* denotes the correlation coefficient between baseline and final measurements; in cases where only standard errors (SEs), 95% confidence intervals (CIs), or interquartile ranges (IQRs) were available, they were converted to SDs using the method proposed by Hozo et al. (Hozo et al. 2005).

A random‐effects model using the DerSimonian‐Laird method (DerSimonian and Laird 1986) was applied to account for heterogeneity across studies and estimate pooled effect sizes. Between‐study heterogeneity was assessed using Cochran's Q statistic and quantified using the *I* ^2^ index (Higgins et al. 2003). An *I* ^2^ value above 40% or a *Q*‐test *p* < 0.05 was considered indicative of substantial heterogeneity (Higgins and Thompson 2002). In addition to the overall pooled analyses, predefined subgroup analyses were performed to explore potential sources of heterogeneity. Subgroups were stratified according to baseline lipid concentrations (e.g., TG < 150 vs. ≥ 150 mg/dL; TC < 200 vs. ≥ 200 mg/dL; LDL‐C < 130 vs. ≥ 130 mg/dL; HDL‐C < 50 vs. ≥ 50 mg/dL), study design (cross‐over vs. parallel), intervention duration (< 12 vs. ≥ 12 weeks), walnut dose (≥ 50 vs. < 50 g/day), type of control group (habitual diet vs. active dietary comparator), participants' health status (healthy vs. at cardiovascular risk), and baseline BMI categories (normal weight, overweight, and obesity).

Non‐linear associations between walnut dose (grams per day) and intervention duration (weeks) with outcomes were examined using fractional polynomial modeling. Additionally, meta‐regression analyses were conducted to investigate linear relationships and the impact of covariates, including sample size, intervention duration, and walnut dosage, on effect estimates (Mitchell 2012).

Publication bias was evaluated using Egger's regression asymmetry test, complemented by visual inspection of funnel plots (Egger et al. 1997). To evaluate the robustness of the pooled estimates, we conducted sensitivity analyses by iteratively removing each individual study (“leave‐one‐out” analysis) for all lipid and apolipoprotein outcomes. All statistical analyses were conducted using STATA software, version 11.2 (StataCorp, College Station, TX, USA). A two‐tailed *p*‐value below 0.05 was considered statistically significant.

### 2.7 Certainty Assessment

The overall quality of the evidence was assessed using the GRADE (Grading of Recommendations Assessment, Development and Evaluation) approach (Gordon et al. 2008). Based on this framework, the certainty of evidence was classified as high, moderate, low, or very low, depending on factors such as study limitations, inconsistency, indirectness, imprecision, and publication bias.

## 3 Results

### 3.1 Study Selection

**FIGURE 1:**
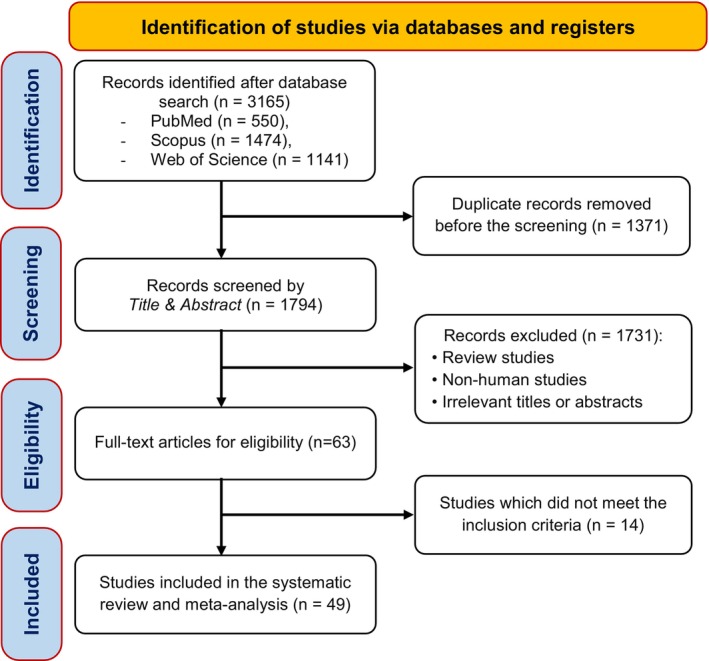
PRISMA flow chart of the study selection process in the systematic review.

As depicted in Figure 1, the initial database search retrieved 3165 records: 550 from PubMed, 1141 from Web of Science, and 1474 from Scopus. After removing 1371 duplicates and screening titles and abstracts, 1731 articles were excluded for failing to meet the inclusion criteria. A full‐text review of the remaining 63 articles resulted in the exclusion of 14 studies due to incomplete or insufficient data. Ultimately, 49 studies met the inclusion criteria and were incorporated into the final meta‐analysis.

### 3.2 Study Characteristic

This meta‐analysis included data from 49 RCTs (Tapsell et al. 2017, 2004, 2009; Hwang et al. 2019; Bashan and Bakman 2018; Sabate et al. 1993; Chisholm et al. 1998; Zambón et al. 2000; Almario et al. 2001; Iwamoto et al. 2002; Morgan et al. 2002; Ros et al. 2004; Zhao et al. 2004; Zibaeenezhad et al. 2005, 2017; Schutte et al. 2006; Canales et al. 2007, 2011; Mukuddem‐Petersen et al. 2007; Perez‐Martinez et al. 2007; Olmedilla‐Alonso et al. 2008; Spaccarotella et al. 2008; Rajaram et al. 2009; Ma Yingying et al. 2010; Torabian et al. 2010; Wu et al. 2010, 2014; Damasceno et al. 2011; Din et al. 2011; Kalgaonkar et al. 2011; Aronis et al. 2012; Katz et al. 2012; Sánchez‐Muniz et al. 2012; Burns‐Whitmore et al. 2014; Müllner et al. 2014; Njike et al. 2015; Bamberger et al. 2017; Rock et al. 2017; Gepner et al. 2018; Holscher et al. 2018; Fatahi et al. 2019; Sanchis et al. 2019; Tindall et al. 2019; Tuccinardi et al. 2019; Al Abdrabalnabi et al. 2020; Kamoun et al. 2021; Gil‐Zamorano et al. 2022; Herselman et al. 2022; Bell et al. 2025), contributing 56 effect sizes and encompassing a total of 4591 participants (2299 in the intervention groups and 2292 in the control groups). The publication dates of these trials ranged from 1993 to 2022. The duration of interventions varied widely across studies, from as short as 4 days to as long as 104 weeks. Of the included studies, 19 utilized a parallel‐group RCT design, and 29 followed a crossover design. Five studies enrolled only male participants, two enrolled only female participants, and the remainder enrolled both sexes. Detailed characteristics of the included studies are presented in Table 1. The results of the risk‐of‐bias assessment are summarized in Table 2.

**TABLE 1:** Characteristics of the included studies in the systematic review and meta‐analysis.

Study	Country	Study design	Health status	Sex	Sample size		Trial duration (week)	Means age		Means BMI		Intervention			Outcomes
					IG	CG		IG	CG	IG	CG	Intervention type	Intervention dose (g/day)	Control	
Sabate et al. (1993)	USA	R/SB/C/CO	Healthy	M	18	18	4	30	30	23.8	23.8	Walnut	84	Control diet	↔TG, TC, LDL, HDL, ApoB ↓ ↑
Chisholm et al. (1998)	New Zealand	R/CO/C	Moderately hyperlipidemic	M	21	21	4	45	45	NR	NR	Diet rich in walnuts	78	Low‐fat diet	↔ TG, TC, LDL, HDL, ApoA1 ↓ ↑
Almario et al. (2001)	USA	R/C	Healthy	M. F	18	18	6	60	60	29	29	Walnuts	48	Habitual diet	↔ TG, TC, LDL, ApoA1, ApoB ↓ HDL ↑
Almario et al. (2001)	USA	R/C	Healthy	M. F	18	18	6	60	60	28.7	27.7	Walnuts + low‐fat diet	48	Low‐fat diet	↔ TG, TC, LDL, HDL, ApoA1, ApoB ↓ ↑
Zambón et al. (2000)	Spain	R/CO/C	Hypercholesterolemic	M. F	49	49	6	56	56	27	27	Walnuts	41–56	cholesterol‐lowering Mediterranean diet	↔ TG, HDL, ApoA1 ↓ TC, LDL, ApoB ↑
Iwamoto et al. (2002)	Japan	R/C/CO	Healthy	M. F	20	20	4	23.8	23.8	22.2	22.32	Walnuts	44–58	Reference diet	↔ TG, TC, LDL, HDL, ApoA1 ↓ ApoB ↑
Iwamoto et al. (2002)	Japan	R/C/CO	Healthy	M. F	20	20	4	23.6	23.6	20.7	20.7	Walnuts	44–58	Reference diet	↔ TG, TC, LDL, HDL, ApoA1 ↓ ApoB ↑
Morgan et al. (2002)	USA	R/CO/C/SB	Hypercholesterolemia	M. F	42	42	6	55.7	55.7	27.7	27.7	Walnut Consumption	64	low‐fat, low‐cholesterol diet	↔ TG, TC, LDL, HDL ↓ ↑
Ros et al. (2004)	Spain	R/CO/C	Dyslipidemia	M. F	20	20	4	25–75	25–75	NR	NR	Walnut Diet	40–65	cholesterol‐lowering Mediterranean diet	↔ TG, HDL, ApoA1, ApoB ↓ TC, LDL ↑
Zhao et al. (2004)	USA	R/C/CO	Hypercholesterolemia	M. F	23	23	6	49.8	49.8	28.1	28.1	Walnuts and walnut oil	100	average American diet	↔ HDL, ApoA1, ApoB ↓TG, TC ↑
Tapsell et al. (2004)	Australia	R/C/P	Type 2 diabetes	M. F	17	21	26	59.3	60.4	30.1	29.2	Walnuts	30 g	modified low‐fat	↔ TG, LDL, HDL ↓ TC ↑
Zibaeenezhad et al. (2005)	Iran	R/C/P	Hyperlipidemia	M. F	23	20	8	NR	NR	NR	NR	Walnuts	20	Control diet	↔ HDL ↓TG, TC, LDL ↑
Schutte et al. (2006)	South Africa	R/C/P	MetS	M. F	20	21	8	45.5	44.4	35.9	35.5	Walnuts	63–108	Control diet	↔ TG ↓ HDL ↑
Perez‐Martinez et al. (2007)	Spain	R/C/CO	Healthy	M	16	16	4	NR	NR	NR	NR	walnuts	NR	Western diet	↔ TG, HDL, ApoA1 ↓ TC, LDL, ApoB ↑
Mukuddem‐Petersen et al. (2007)	South Africa	R/C/P	MetS	M. F	21	22	8	45	45	36	35.1	High walnut diet	63–108	nut‐free diet	↔ TG, TC, LDL ↓ HDL ↑
Canales et al. (2007)	SPAIN	R/CO/C	Overweight/Obese Senior Subjects with at Least One Extra CHD‐Risk Factor	M. F	22	22	5	54.8	54.8	29.6	29.6	Meat enriched with walnuts	21.42	meat without walnut	↔ ↓ ↑ HDL
Spaccarotella et al. (2008)	USA	R/CO/C	Healthy elderly	M	21	21	8	65.9	65.9	27.5	27.5	walnuts	75	Usual diet	↔ TG, TC, LDL, HDL ↓ ↑
Olmedilla‐Alonso et al. (2008)	SPAIN	R/CO/C	at risk for cardiovascular disease	M. F	25	25	5	54.4	54.4	30	30	Walnuts	19.4	regular consumption of the meat product without walnuts	↔ TG, TC, LDL, HDL ↓ ↑
Rajaram et al. (2009)	USA	R/C/CO	Hyperlipidemia	M. F	25	25	4	23–65	23–65	24.8	24.8	Walnuts	42.5	control diet	↔ TG, HDL, ApoA1, ApoB ↓ TC, LDL ↑
Tapsell et al. (2009)	Australia	R/C/P	Type 2 diabetes	M. F	18	17	51	54	54	33.2	33	Walnuts	30	low‐fat isocaloric dietary advice	↔ TG, TC, LDL, HDL ↓ ↑
Wu et al. (2010)	China	R/C/P	MetS	M. F	94	95	12	48.2	48.6	25.7	25.4	Walnuts	30	Lifestyle counseling without walnuts	↔ TG, TC, LDL, HDL, ApoA1, ApoB ↓ ↑
Torabian et al. (2010)	USA	R/CO/C	Normal to moderately high plasma total cholesterol	M. F	87	87	26	54	54	26.5	26.5	walnuts	28–64	Habitual diet	↔ TG, TC, HDL ↓ LDL ↑
Ma et al. (2010)	USA	R/CO/C/SB	Type 2 diabetes	M. F	21	21	8	58.1	58.1	32.5	32.5	walnut‐enriched ad libitum	56	ad libitum diet without walnuts	↔ TG, TC, LDL, HDL ↓ ↑
Damasceno et al. (2011)	Spain	R/C/CO	Moderate hypercholesterolemia	M. F	18	18	4	56	56	25.7	25.7	Walnut Diet	40–65	Virgin olive oil	↔ TG, TC, LDL, HDL, ApoA1, ApoB ↓ ↑
Din et al. (2011)	UK	R/CO/C/SB	Healthy	M	30	30	4	23	23	24.5	24.5	Moderate walnut consumption	15	Walnut‐excluded diet	↔ TG, TC, LDL, HDL ↓ ↑
Canales et al. (2011)	Spain	R/C/CO	At high risk for CVD	M. F	22	22	5	54.8	54.8	29.6	29.6	Meat enriched with walnuts	21	Meat without walnut	↔ HDL, ApoA1 ↓ ↑
Kalgaonkar et al. (2011)	USA	R/C	PCOS	F	17	14	6	31.2	36.2	35.2	35.1	Walnuts	31	Almond	↔ TG, TC, LDL, HDL, ApoB ↓ ↑
Aronis et al. (2012)	USA	R/dB/P/CO	MetS	M. F	15	15	0.57	58	58	36.6	36.6	Walnuts	48	Control group	↔ ApoA1, ApoB ↓ ↑
Katz et al. (2012)	USA	R/CO/C/SB	Overweight and obese adults	M. F	40	40	8	57.4	57.4	33.2	33.2	Walnut‐Enriched Diet	56	ad libitum diet without walnuts	↔ TC, LDL, HDL ↓ ↑
Sánchez‐Muniz et al. (2012)	Spain	R/C/CO	At high risk for CVD (QQ)	M. F	11	11	5	55	55	30	29.8	Low‐fat meat with walnuts	8/5	Low‐fat meat without walnuts	↔ TG, TC, LDL, HDL ↓ ↑
Sánchez‐Muniz et al. (2012)	Spain	R/C/CO	At high risk for CVD (QR + RR)	M. F	11	11	5	55	55	29	29.1	Low‐fat meat with walnuts	8/5	Low‐fat meat without walnuts	↔ TG, TC, LDL, HDL ↓ ↑
Sánchez‐Muniz et al. (2012)	Spain	R/C/CO	At high risk for CVD (LL)	M. F	8	8	5	55	55	29.3	29.7	Low‐fat meat with walnuts	8/5	Low‐fat meat without walnuts	↔ TG, LDL, HDL ↓ ↑ TC
Sánchez‐Muniz et al. (2012)	Spain	R/C/CO	At high risk for CVD (LM + MM)	M. F	14	14	5	55	55	29.7	29.4	Low‐fat meat with walnuts	8/5	Low‐fat meat without walnuts	↔ TG, TC, LDL, HDL ↓ ↑
Müllner et al. (2014)	Austria	R/dB	Insulin‐treated T2DM	M. F	18	16	10	63	63	NR	NR	Walnut Oil	9	Mixed oil	↔ TG, TC, LDL, HDL ↓ ↑
Müllner et al. (2014)	Austria	R/dB	T2DM	M. F	29	29	10	62.3	62.3	NR	NR	Walnut Oil	9	Mixed oil	↔ TG, TC, LDL, HDL ↓ ↑
Wu et al. (2014)	Germany	R/CO/C	Healthy caucasian	M. F	40	40	8	60	60	24.9	24.9	Walnut‐enriched diet	43	Western Diet	↔ TG, TC, LDL, HDL, ApoB ↓ ↑
Burns‐Whitmore et al. (2014)	USA	R/CO/C	Healthy free‐living lacto‐ovo‐vegetarians	M. F	20	20	8	38	38	23	23	Walnuts	28.4	Standard egg	↔ TG, TC, LDL, HDL, ApoA1, ApoB ↓ ↑
Njike et al. (2015)	USA	R/C/P	At risk for diabetes	M. F	26	26	26	56.5	56.5	30	30	Walnut Consumption	56	Calorie‐adjusted diet	↔ TG, TC, LDL, HDL ↓ ↑
Njike et al. (2015)	USA	R/C/P	At risk for diabetes	M. F	26	23	26	53.3	53.3	30.2	30.2	Walnut Consumption	56	ad libitum diet	↔TG, TC, LDL, HDL ↓ ↑
Bamberger et al. (2017)	Germany	R/CO/C	Healthy	M. F	194	194	8	63	63	25.1	25.1	Walnut‐enriched diet	43	Nut‐free diet	↔ HDL, ApoB ↓ TG, TC, LDL ↑
Zibaeenezhad et al. (2017)	Iran	R/C/dB	Hyperlipidemic T2DM	M. F	45	45	12	55.5	54	27.6	27.2	Walnut Oil	15	Placebo	↔ HDL ↓ TG, TC, LDL ↑
Rock et al. (2017)	USA	R/C/P	Non‐diabetic overweight and obese	M. F	49	51	24	5.3	52.2	32.4	32.4	Walnut‐enriched reduced‐energy diet	42	Standard reduced‐energy diet	↔ TC, LDL ↓ TG ↑ HDL
Tapsell et al. (2017)	Australia	R/C	Overweight or obese	M. F	99	103	12	45	45	32	32	Interdisciplinary protocol + a healthy food supplement (walnuts)	30	Interdisciplinary protocol	↔ ↓ TG, TC, LDL ↑ HDL
Holscher et al. (2018)	USA	R/C/CO	Healthy	M. F	18	18	3	53	53.1	28.8	28.8	Walnuts	42	Control group	↔ TC, LDL, HDL ↓ ↑ TG
Bashan and Bakman (2018)	Turkey	R/C	Dyslipidemia	M. F	73	72	8	41.0	40.7	30.9	30.1	Walnut consumption in regulated diet	40–50	Regulated diet	↔ ↓ TG, TC, LDL ↑ HDL
Gepner et al. (2018)	Israel	R/C/CO	Sedentary adults	M. F	56	58	24	48	48	30.8	30.8	MED/LC diet + walnuts	28	Low‐fat	↔ TG, TC, HDL ↓ ↑
Gepner et al. (2018)	Israel	R/C/CO	Sedentary adults	M. F	60	66	24	48	48	30.8	30.8	MED/LC diet + walnuts + moderate PA	28	Low‐fat + moderate PA	↔ TC ↓ TG ↑ HDL
Tindall et al. (2019)	USA	R/CO/C	At high risk for CVD	M. F	36	36	6	43	43	30.3	30.3	Walnuts	57–99	walnut FA‐matched diet	↔ TG, TC, LDL ↓ ↑
Tuccinardi et al. (2019)	Italy	R/C/CO	Obesity	M. F	10	10	0.71	50.7	50.7	36.8	36.8	Walnuts	48	Placebo	↔ TG, TC, LDL, HDL ↓ ↑
Fatahi et al. (2019)	Iran	R/C/P	Overweight and Obese Women	F	33	33	12	54.5	52.9	33.5	32.9	Walnut + Fish	2.57	Fish	↔ TG, LDL ↓ ↑ HDL
Sanchis et al. (2019)	Spain	R/C/CO	Chronic Kidney Disease	M. F	13	13	4	71	71	27	32	Walnut diet	30	Control diet	↔ TG, TC, LDL, HDL ↓ ↑
Hwang et al. (2019)	Korea	R/CO/C	MetS	M. F	84	84	16	41	37.9	27.9	26.2	Walnut	45	iso‐caloric white bread	↔ TC, HDL, ApoB ↓ ↑ TG, LDL
Kamoun et al. (2021)	Tunisia	R/C	Healthy	M	10	10	6	66.5	66.9	24.5	25.5	Walnut + Training	15	Training	↔ LDL ↓ TG, TC ↑ HDL
Al Abdrabalnabi et al. (2020)	USA	R/C/Cohort	Healthy elderly	M. F	319	306	104	69.2	69.1	27.1	27.5	Walnuts	30–60	Habitual diet	↔ TG, HDL ↓ ↑
Herselman et al. (2022)	Australia	R/C/P	Healthy	M. F	30	30	16	22	22	22.8	22.8	Walnuts	56	Control group	↔ ↓ TG ↑ TC
Gil‐Zamorano et al. (2022)	Spain	R/C/B/P	Healthy elderly	M. F	166	164	52	69	68.8	26.8	27.4	Walnuts	30–60	Control group	↔ TG, HDL ↓ TC, LDL ↑
Mates et al. (2025)	Romania	R/C	Middle‐Aged Adults	M. F	11	9	4	49	47.6	28.1	30	Walnuts	45	Control group	↔ TG, TC, LDL, HDL ↓ ↑

*Note:* Some studies contributed more than one effect size/intervention arm, and the total number of unique RCTs included in the meta‐analysis is 49, comprising 57 effect sizes.

Abbreviations: CG, control group; CO, controlled; CVD, cardiovascular disease; DB, double‐blinded; F, female; IG, intervention group; M, male; MED/LC, Mediterranean/low‐carbohydrate diet; MetS, Metabolic syndrome; NR, not reported; PA, physical activity; PC, placebo‐controlled; PCOS, polycystic ovary syndrome; R, randomized; SB, single‐blinded; T2DM, type 2 diabetes mellitus.

**TABLE 2:** Risk of bias (RoB) assessment of included studies.

Studies	D1	D2	D3	D4	D5	Overall RoB
Sabate et al. (1993)	L	H	L	H	L	High‐risk of bias
Chisholm et al. (1998)	L	H	L	H	L	High‐risk of bias
Zambón et al. (2000)	L	H	L	H	L	High‐risk of bias
Almario et al. (2001)	L	H	L	H	L	High‐risk of bias
Almario et al. (2001)	L	H	L	H	L	High‐risk of bias
Iwamoto et al. (2002)	L	H	L	H	L	High‐risk of bias
Iwamoto et al. (2002)	L	H	L	H	L	High‐risk of bias
Morgan et al. 2002	L	H	L	H	L	High‐risk of bias
Ros et al. (2004)	L	H	L	H	L	High‐risk of bias
Tapsell et al. (2004)	L	H	H	H	L	High‐risk of bias
Zhao et al. (2004)	L	H	L	H	L	High‐risk of bias
Zibaeenezhad et al. (2005)	L	H	H	H	L	High‐risk of bias
Schutte et al. (2006)	L	H	H	H	H	High‐risk of bias
Canales et al. (2007)	L	H	L	H	H	High‐risk of bias
Mukuddem‐Petersen et al. (2007)	L	H	H	H	L	High‐risk of bias
Perez‐Martinez et al. (2007)	L	H	L	H	L	High‐risk of bias
Olmedilla‐Alonso et al. (2008)	L	H	L	H	L	High‐risk of bias
Spaccarotella et al. (2008)	L	H	L	H	L	High‐risk of bias
Rajaram et al. (2009)	L	H	L	H	L	High‐risk of bias
Tapsell et al. (2009)	L	H	H	H	L	High‐risk of bias
Ma et al. (2010)	L	H	L	H	L	High‐risk of bias
Torabian et al. (2010)	L	H	L	H	L	High‐risk of bias
Wu et al. (2010)	L	H	H	H	L	High‐risk of bias
Canales et al. (2011)	L	H	L	H	H	High‐risk of bias
Damasceno et al. (2011)	L	H	L	H	L	High‐risk of bias
Din et al. (2011)	L	H	L	H	L	High‐risk of bias
Kalgaonkar et al. (2011)	L	H	H	H	H	High‐risk of bias
Aronis et al. (2012)	L	L	L	H	H	High‐risk of bias
Katz et al. (2012)	L	H	L	H	L	High‐risk of bias
Sánchez‐Muniz et al. (2012)	L	H	L	H	L	High‐risk of bias
Sánchez‐Muniz et al. (2012)	L	H	L	H	L	High‐risk of bias
Sánchez‐Muniz et al. (2012)	L	H	L	H	L	High‐risk of bias
Sánchez‐Muniz et al. (2012)	L	H	L	H	L	High‐risk of bias
Burns‐Whitmore et al. (2014)	L	H	L	H	L	High‐risk of bias
Müllner et al. (2014)	L	L	H	H	L	High‐risk of bias
Müllner et al. (2014)	L	H	L	H	L	High‐risk of bias
Wu et al. (2014)	L	H	L	H	H	High‐risk of bias
Njike et al. (2015)	L	H	L	H	L	High‐risk of bias
Njike et al. (2015)	L	H	H	H	L	High‐risk of bias
Rock et al. (2017)	L	H	H	H	H	High‐risk of bias
Bamberger et al. (2017)	L	H	L	H	H	High‐risk of bias
Rock et al. (2017)	L	H	H	H	L	High‐risk of bias
Tapsell et al. (2017)	L	H	H	H	L	High‐risk of bias
Zibaeenezhad et al. (2017)	L	L	L	H	L	Some concerns
Bashan and Bakman (2018)	L	H	H	H	L	High‐risk of bias
Gepner et al. (2018)	L	H	H	H	H	High‐risk of bias
Gepner et al. (2018)	L	H	H	H	H	High‐risk of bias
Holscher et al. (2018)	L	H	L	H	L	High‐risk of bias
Fatahi et al. (2019)	L	H	L	H	H	High‐risk of bias
Hwang et al. (2019)	L	H	L	H	H	High‐risk of bias
Sanchis et al. 2019	L	H	L	H	L	High‐risk of bias
Tindall et al. (2019)	L	H	L	H	L	High‐risk of bias
Tuccinardi et al. (2019)	L	H	L	H	L	High‐risk of bias
Al Abdrabalnabi et al. (2020)	L	H	H	H	H	High‐risk of bias
Kamoun et al. (2021)	L	H	L	H	L	High‐risk of bias
Gil‐Zamorano et al. (2022)	L	H	H	H	L	High‐risk of bias
Herselman et al. (2022)	L	H	L	H	H	High‐risk of bias
Mates et al. (2025)	L	H	H	H	L	High‐risk of bias

*Note:* some studies contributed more than one effect size/intervention arm, and the total number of unique RCTs included in the meta‐analysis is 49. Domains: D1, Bias arising from the randomization process; D2, Bias due to deviations from intended interventions; D3, Bias due to missing outcome data; D4, Bias in measurement of the outcome; D5, Bias in selection of the reported result. Interpretation: L, low‐risk of bias; H, high‐risk of bias; General risk of bias, High‐risk of bias > 2H; Some concerns = 1H.

### 3.3 Effects of Walnut Consumption on TG

**FIGURE 2:**
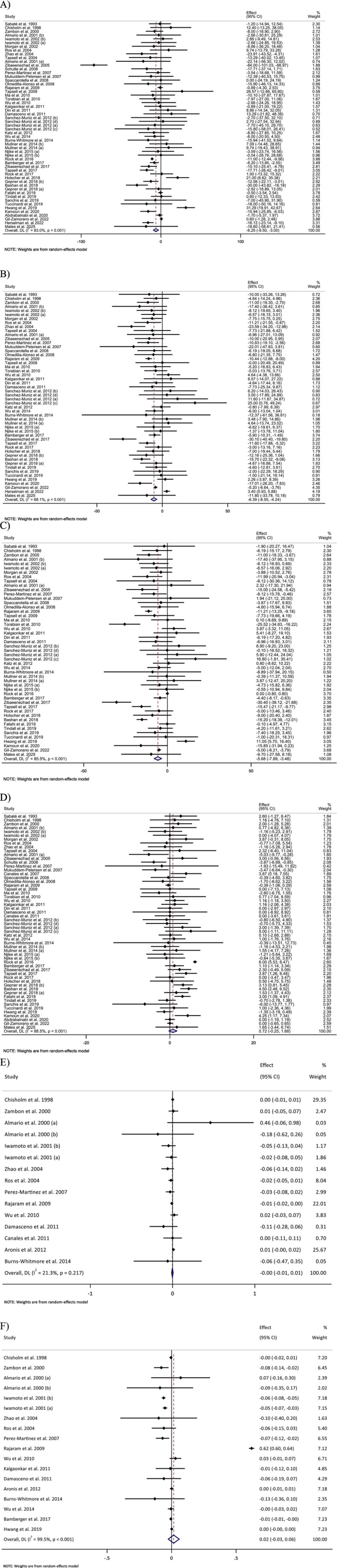
Forest plot detailing weighted mean difference and 95% confidence intervals (CIs) for the effect of walnut consumption on (A) TG (mg/dL); (B) TC (mg/dL); (C) LDL (mg/dL); (D) HDL (mg/dL); (E) Apo A1 (g/L); (F) Apo B (g/L).

A meta‐analysis of 53 effect sizes evaluating TG showed a statistically significant reduction following walnut intake (WMD: −6.25 mg/dL; 95% CI: −9.50 to −3.00; *p* < 0.001) (Figure 2A). However, substantial heterogeneity was observed among the included studies (*I* ^2^ = 83.0%). Subgroup analyses revealed that walnut consumption did not significantly influence TG levels in individuals with baseline TG < 150 mg/dL, in crossover‐designed trials, in studies with intervention durations of ≥ 12 weeks, in participants with normal or overweight BMI, or in comparisons involving active controls (Table 3).

**TABLE 3:** Subgroup analyses of walnut consumption on lipid profile and apolipoproteins in adults.

	No. of ES	WMD (95% CI)	*p*	Heterogeneity	
				P‐heterogeneity	*I* ^2^
Triglycerids (TG: mg/dL)					
Overall effect	55	−6.24 (−9.49, −2.99)	**< 0.001**	< 0.001	83.0%
Baseline TG (mg/dL)					
< 150	34	−3.20 (−7.02, 0.02)	0.052	< 0.001	70.4%
> 150	18	−12.48 (−23.21, −1.76)	**0.022**	< 0.001	85.3%
Study design					
Cross‐over	31	−1.79 (−6.13, 2.53)	0.416	< 0.001	56.4%
Parallel	24	−11.03 (−15.85, −6.20)	**< 0.001**	< 0.001	90.2%
Trial duration (week)					
< 12	37	−7.55 (−12.84, −2.26)	**0.005**	< 0.001	73.6%
≥ 12	18	−4.59 (−9.27, 0.09)	0.055	< 0.001	90.4%
Dose (g/day)					
≥ 50	18	−7.28 (−11.42, −3.14)	**0.001**	0.113	29.8%
< 50	36	−6.59 (−10.74, −2.43)	**0.002**	< 0.001	82.9%
Control group					
Habitual diet	43	−7.32 (−11.13, −3.52)	**< 0.001**	< 0.001	85.7%
Other intervention	12	−2.88 (−7.49, 1.72)	0.220	0.254	19.3%
Health status					
Healthy	20	−4.85 (−8.77, −0.93)	**0.015**	< 0.001	65.8%
At CVD risk	35	−7.32 (−12.19, −2.46)	**0.003**	< 0.001	84.3%
Baseline BMI (kg/m^2^)					
Normal (18.5–24.9)	9	−6.21 (−12.53, 0.10)	0.054	0.002	66.5%
Overweight (25–29.9)	20	−1.75 (−6.85, 3.33)	0.498	< 0.001	71.7%
Obese (≥ 30)	19	−8.38 (−13.75, −3.00)	**0.002**	0.003	54.1%
Total Cholesterol (TC: mg/dL)					
Overall effect	51	−6.39 (−8.54, −4.23)	**< 0.001**	< 0.001	68.1%
Baseline TC (mg/dL)					
< 200	21	−4.86 (−9.01, −0.71)	**0.022**	< 0.001	77.7%
> 200	27	−7.44 (−10.18, −4.71)	**< 0.001**	< 0.001	58.9%
Study design					
Cross‐over	31	−6.30 (−8.49, −4.11)	**< 0.001**	0.037	33.7%
Parallel	20	−6.98 (−11.02, −2.94)	**0.001**	< 0.001	81.0%
Trial duration (week)					
< 12	36	−7.58 (−9.75, −5.41)	**< 0.001**	0.033	32.5%
≥ 12	15	−4.94 (−8.97, −0.92)	**0.016**	< 0.001	81.7%
Dose (g/day)					
≥ 50	16	−6.38 (−10.57, −2.18)	**0.003**	< 0.001	67.1%
< 50	34	−6.49 (−8.97, −4.01)	**< 0.001**	< 0.001	60.9%
Control group					
Habitual diet	40	−6.56 (−8.98, −4.15)	**< 0.001**	< 0.001	73.6%
Other intervention	11	−6.26 (−10.33, −2.19)	**0.003**	0.522	0.0%
Health status					
Healthy	19	−6.39 (−9.47, −3.32)	**< 0.001**	< 0.001	66.4%
At CVD risk	32	−6.00 (−9.07, −2.94)	**< 0.001**	< 0.001	63.9%
Baseline BMI (kg/m^2^)					
Normal (18.5–24.9)	9	−7.05 (−13.55, −0.55)	**0.033**	< 0.001	88.6%
Overweight (25–29.9)	19	−6.68 (−10.41, −2.95)	**< 0.001**	< 0.001	67.9%
Obese (≥ 30)	17	−6.52 (−9.68, −3.37)	**< 0.001**	0.207	21.2%
Low‐Density Lipoprotein Cholesterol (LDL‐C: mg/dL)					
Overall effect	49	−5.68 (−7.89, −3.47)	**< 0.001**	< 0.001	85.5%
Baseline LDL‐C (mg/dL)					
< 130	26	−4.20 (−7.00, −1.40)	**0.003**	< 0.001	74.3%
> 130	19	−8.23 (−12.54, −3.93)	**< 0.001**	< 0.001	79.7%
Study design					
Cross‐over	28	−4.93 (−7.97, −1.88)	**0.002**	< 0.001	72.7%
Parallel	21	−6.64 (−9.97, −3.30)	**< 0.001**	< 0.001	89.7%
Trial duration (week)					
< 12	35	−6.13 (−8.35, −3.92)	**< 0.001**	< 0.001	51.2%
≥ 12	14	−5.66 (−9.34, −1.98)	**0.003**	< 0.001	92.0%
Dose (g/day)					
≥ 50	15	−3.06 (−5.36, −0.76)	**0.009**	0.195	23.4%
< 50	33	−6.36 (−9.27, −3.46)	**< 0.001**	< 0.001	83.3%
Control group					
Habitual diet	39	−5.89 (−8.37, −3.41)	**< 0.001**	< 0.001	88.1%
Other intervention	10	−4.71 (−8.77, −0.66)	**0.023**	0.159	31.2%
Health status					
Healthy	16	−7.33 (−10.00, −4.66)	**< 0.001**	0.066	37.4%
At CVD risk	33	−4.42 (−7.58, −1.25)	**0.006**	< 0.001	88.8%
Baseline BMI (kg/m^2^)					
Normal (18.5–24.9)	8	−10.31 (−12.13, −8.50)	**< 0.001**	0.589	0.0%
Overweight (25–29.9)	18	−5.66 (−9.97, −1.35)	**0.010**	< 0.001	83.8%
Obese (≥ 30)	16	−4.35 (−8.64, −0.06)	**0.046**	< 0.001	71.9%
High‐Density Lipoprotein Cholesterol (HDL‐C: mg/dL)					
Overall effect	56	0.71 (−0.24, 1.68)	0.145	< 0.001	88.5%
Baseline HDL‐C (mg/dL)					
< 50	26	0.92 (−0.15, 1.99)	0.092	< 0.001	64.1%
> 50	27	0.43 (−0.09, 0.94)	0.098	0.392	4.9%
Study design					
Cross‐over	33	0.25 (−0.28, 0.80)	0.353	0.376	5.6%
Parallel	23	0.98 (−0.62, 2.59)	0.232	< 0.001	93.4%
Trial duration (week)					
< 12	39	0.43 (−0.33, 1.21)	0.267	0.001	44.8%
≥ 12	17	1.38 (−0.44, 3.20)	0.138	< 0.001	94.7%
Dose (g/day)					
≥ 50	17	−0.10 (−2.50, 2.28)	0.929	< 0.001	91.1%
< 50	38	1.10 (0.46, 1.75)	**0.001**	< 0.001	50.8%
Control group					
Habitual diet	44	0.57 (−0.54, 1.68)	0.314	< 0.001	90.9%
Other intervention	12	1.90 (0.89, 2.92)	**< 0.001**	0.794	0.0%
Health status					
Healthy	19	0.61 (−0.08, 1.31)	0.083	0.241	17.4%
At CVD risk	37	0.71 (−0.59, 2.03)	0.284	< 0.001	90.5%
Baseline BMI (kg/m^2^)					
Normal (18.5–24.9)	8	0.64 (−0.64, 1.93)	0.326	0.136	36.7%
Overweight (25–29.9)	22	0.33 (−0.33, 0.99)	0.331	0.239	16.6%
Obese (≥ 30)	19	0.65 (−0.58, 1.89)	0.300	< 0.001	64.7%
Apolipoprotein A1 (Apo‐A1: mg/dL)					
Overall effect	15	−0.00 (−0.01, 0.00)	0.573	0.217	21.3%
Study design					
Cross‐over	12	−0.00 (−0.01, 0.00)	0.518	0.267	18.0%
Parallel	3	0.04 (−0.18, 0.28)	0.691	0.174	42.8%
Trial duration (week)					
< 12	14	−0.00 (−0.01, 0.00)	0.446	0.198	23.6%
≥ 12	1	0.02 (−0.02, 0.06)	0.394	—	—
Dose (g/day)					
≥ 50	6	−0.01 (−0.03, 0.00)	0.195	0.225	28.0%
< 50	8	0.00 (−0.01, 0.01)	0.693	0.257	21.7%
Control group					
Habitual diet	10	−0.00 (−0.02, 0.01)	0.794	0.163	29.7%
Other intervention	4	−0.01 (−0.04, 0.01)	0.226	0.559	0.0%
Health status					
Healthy	6	−0.02 (−0.06, 0.00)	0.135	0.526	0.0%
At CVD risk	9	−0.00 (−0.01, 0.00)	0.842	0.182	29.6%
Baseline BMI (kg/m^2^)					
Normal (18.5–24.9)	4	−0.01 (−0.02, 0.00)	0.103	0.838	0.0%
Overweight (25–29.9)	7	−0.00 (−0.05, 0.03)	0.734	0.209	28.7%
Obese (≥ 30)	1	0.01 (−0.00, 0.02)	0.093	—	—
Apolipoprotein B (Apo‐B: mg/dL)					
Overall effect	18	0.01 (−0.02, 0.05)	0.462	< 0.001	99.5%
Study design					
Cross‐over	14	0.01 (−0.03, 0.06)	0.465	< 0.001	99.6%
Parallel	4	0.02 (−0.01, 0.06)	0.262	0.722	0.0%
Trial duration (week)					
< 12	16	0.00 (−0.06, 0.08)	0.836	< 0.001	99.6%
≥ 12	2	0.00 (−0.01, 0.02)	0.566	0.205	37.8%
Dose (g/day)					
≥ 50	6	−0.04 (−0.07, −0.00)	**0.013**	< 0.001	88.6%
< 50	11	0.05 (−0.00, 0.11)	0.052	< 0.001	99.7%
Control group					
Habitual diet	13	0.04 (−0.00, 0.08)	0.112	< 0.001	99.7%
Other intervention	5	−0.06 (−0.10, −0.02)	**0.002**	0.824	0.0%
Health status					
Healthy	8	−0.03 (−0.06, −0.00)	**0.017**	< 0.001	92.1%
At CVD risk	10	0.04 (−0.07, 0.15)	0.458	< 0.001	99.7%
Baseline BMI (kg/m^2^)					
Normal (18.5–24.9)	5	0.08 (−0.19, 0.35)	0.573	< 0.001	99.9%
Overweight (25–29.9)	8	−0.00 (−0.01, 0.00)	0.433	0.008	63.6%
Obese (≥ 30)	2	0.00 (−0.01, 0.01)	0.950	0.854	0.0%

*Note:* Bold values are statistically significant [*p* < 0.05].

Abbreviations: Apo‐1A, apolipoprotein A1; Apo‐B, apolipoprotein B; CI, confidence interval; ES, effect size; HDL‐C, high‐density lipoprotein cholesterol; LDL‐C, low‐density lipoprotein cholesterol; TC, total cholesterol; TG, triglycerides; WMD, weighted mean differences.

### 3.4 Effects of Walnut Consumption on TC

Pooled analysis revealed that walnut intake significantly reduced TC (WMD: −6.39 mg/dL; 95% CI: −8.54 to −4.23; *p* < 0.001) (Figure 2B). Heterogeneity was moderate for TC (*I* ^2^ = 68.1%). The markers consistently decreased across all subgroup analyses.

### 3.5 Effects of Walnut Consumption on LDL‐C

The pooled results demonstrated that walnut consumption led to a statistically significant decrease in LDL‐C levels (WMD: −5.68 mg/dL; 95% CI: −7.89 to −3.47; *p* < 0.001) (Figure 2C). Considerable between‐study heterogeneity was present (*I* ^2^ = 85.5%). Despite this variability, a consistent LDL‐C–lowering effect was observed across all subgroup analyses.

### 3.6 Effects of Walnut Consumption on HDL‐C

Meta‐analysis of 56 effect sizes assessing the impact of walnut consumption on HDL‐C revealed no statistically significant overall effect (WMD: 0.71 mg/dL; 95% CI: −0.24 to 1.68; *p* = 0.145) (Figure 2D). High heterogeneity was detected across studies (*I* ^2^ = 88.5%). However, subgroup analyses indicated a significant elevation in HDL‐C levels in studies in which the control group did not follow a habitual diet and in interventions employing walnut doses below 50 g/day (Table 3).

### 3.7 Effects of Walnut Consumption on Apolipoproteins

Combined data from eligible studies showed that walnut intake had no significant effect on Apo‐A1 (WMD: −0.00 g/L; 95% CI: −0.01 to 0.00; *p* = 0.573) (Figure 2E) or Apo‐B (WMD: 0.01 g/L; 95% CI: −0.02 to 0.05; *p* = 0.462) (Figure 2F). The heterogeneity was low for Apo‐A1 (*I* ^2^ = 23.6%) and high for Apo‐B (*I* ^2^ = 65.6%). However, subgroup analysis showed a significant reduction in Apo‐B levels when walnut intake exceeded 50 g/day (Table 3).

### 3.8 Publication Bias Assessment

**FIGURE 3:**
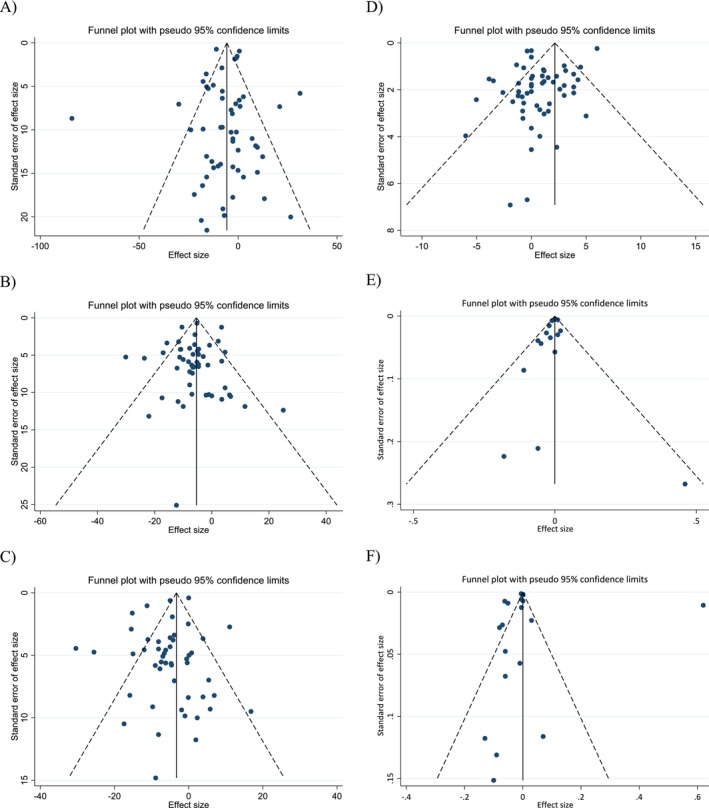
Funnel plots for the effect of walnut consumption on (A) TG (mg/dL); (B) TC (mg/dL); (C) LDL (mg/dL); (D) HDL (mg/dL); (E) Apo A1 (g/L); (F) Apo B (g/L).

Evaluation of funnel plots (Figure 3), along with Egger's and Begg's statistical tests, indicated no substantial evidence of publication bias across the studies investigating the impact of walnut intake on lipid and apolipoprotein outcomes (Table 4).

**TABLE 4:** Publication bias analysis by Begg's and Egger's tests.

Outcomes	Publication bias	
	Begg's test	Egger's test
TG	0.988	0.974
TC	0.262	0.482
LDL‐C	0.057	0.082
HDL‐C	0.329	**0.009**
Apo‐A1	0.921	0.211
Apo‐B	0.495	0.575

*Note:* Bold values are statistically significant [*p* < 0.05].

Abbreviations: Apo‐A1, apolipoprotein A1; Apo‐B, apolipoprotein B; HDL‐C, high‐density lipoprotein cholesterol; LDL‐C, low‐density lipoprotein cholesterol; TC, total cholesterol; TG, triglycerides.

### 3.9 Dose–Response Analyses

**FIGURE 4:**
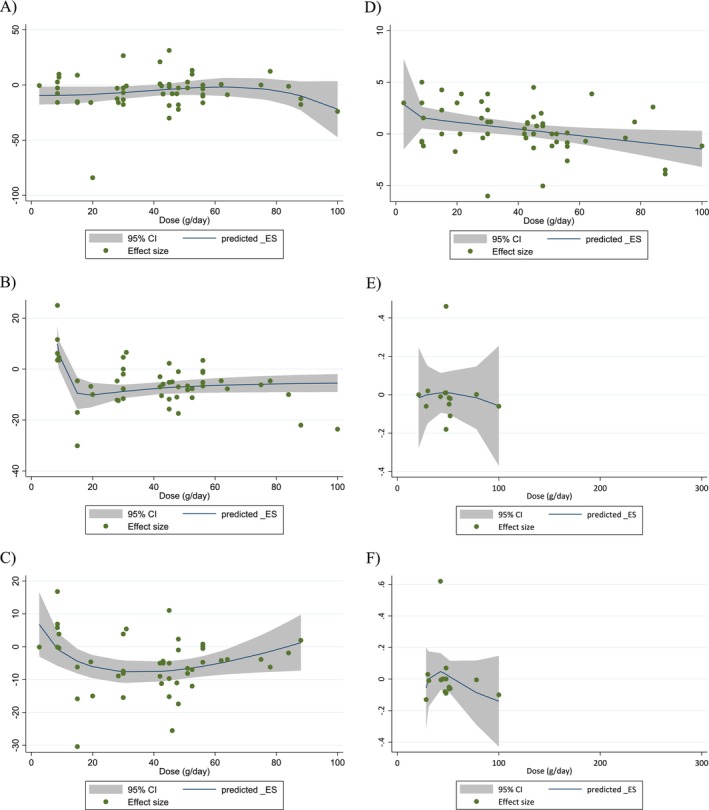
Non‐linear dose–response relations between walnut consumption and absolute mean differences. Dose–response relations between dose (g/day) and absolute mean differences in (A) TG (mg/dL); (B) TC (mg/dL); (C) LDL (mg/dL); (D) HDL (mg/dL); (E) Apo A1 (g/L); (F) Apo B (g/L).

**FIGURE 5:**
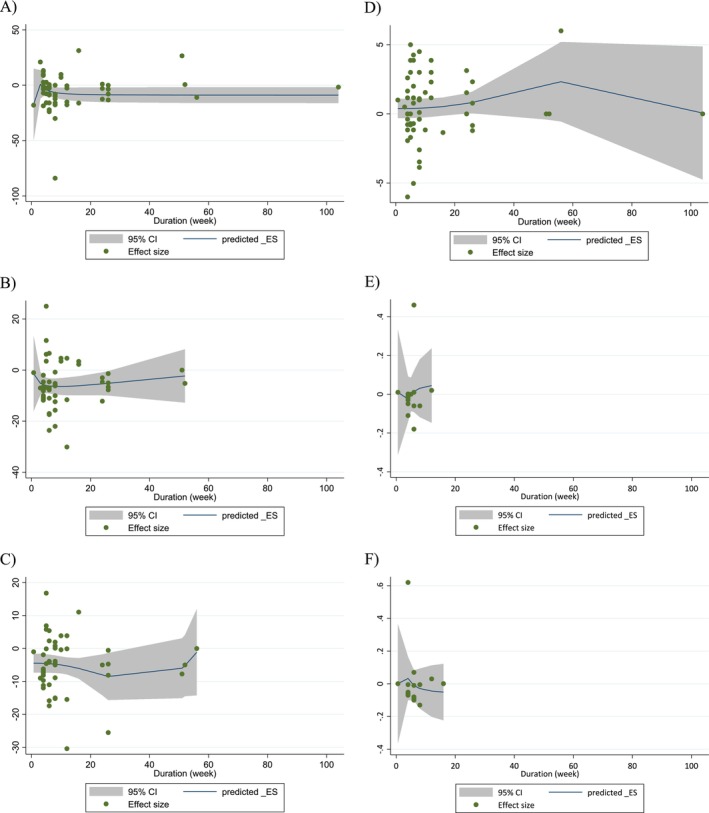
Non‐linear dose–response relations between walnut consumption and absolute mean differences. Dose–response relations between duration of intervention (week) and absolute mean differences in (A) TG (mg/dL); (B) TC (mg/dL); (C) LDL (mg/dL); (D) HDL (mg/dL); (E) Apo A1 (g/L); (F) Apo B (g/L).

**FIGURE 6:**
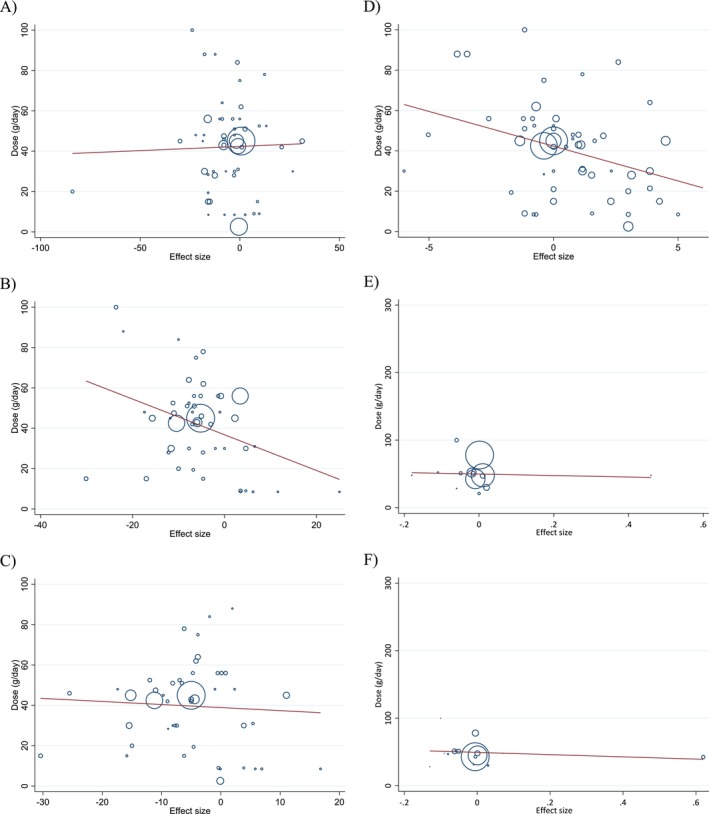
Linear dose–response relations between walnut consumption and absolute mean differences. Dose–response relations between dose (g/day) and absolute mean differences in (A) TG (mg/dL); (B) TC (mg/dL); (C) LDL (mg/dL); (D) HDL (mg/dL); (E) Apo A1 (g/L); (F) Apo B (g/L).

**FIGURE 7:**
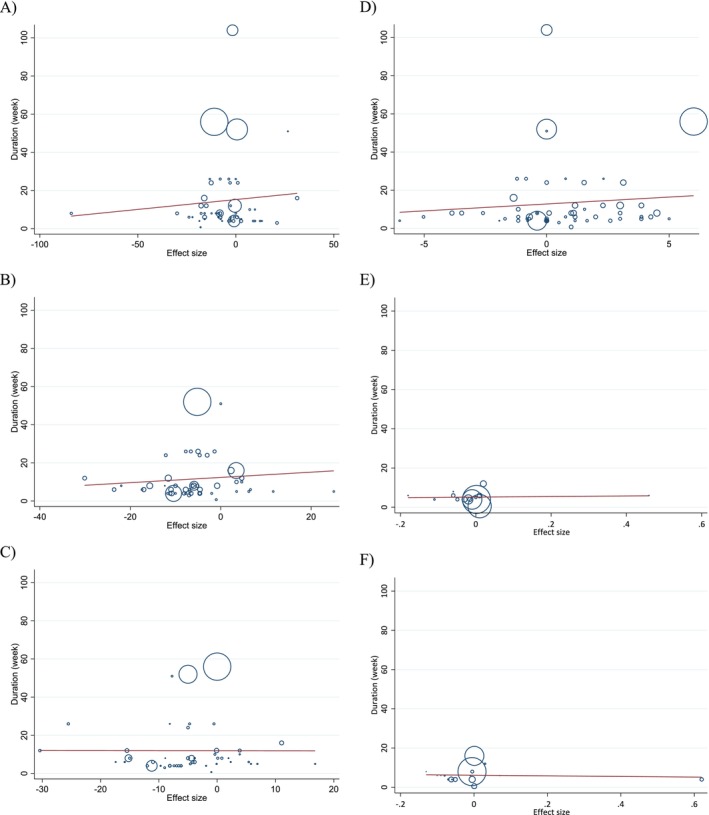
Linear dose–response relations between walnut consumption and absolute mean differences. Dose–response relations between duration of intervention (week) and absolute mean differences in (A) TG (mg/dL); (B) TC (mg/dL); (C) LDL (mg/dL); (D) HDL (mg/dL); (E) Apo A1 (g/L); (F) Apo B (g/L).

Non‐linear dose–response modeling identified significant associations between walnut dose and changes in TC (coefficient: −37.76; *p* = 0.031), LDL‐C (coefficient: −28.81; *p* = 0.013), and HDL‐C (coefficient: −0.31; *p* = 0.028). Similarly, linear regression analyses showed that higher walnut doses were associated with reductions in TC (coefficient: −0.88; *p* = 0.010) and HDL‐C (coefficient: −3.44; *p* = 0.010) (Table 5; Figures 4, 5, 6, 7).

**TABLE 5:** Linear meta regression and non‐linear dose response analysis.

Outcomes	Linear regression				Non‐linear dose response			
	Dose		Duration		Dose		Duration	
	Coefficient	*p*	Coefficient	*p*	Coefficient	*p*	Coefficient	*p*
TG	0.04	0.827	0.10	0.561	−0.09	0.142	−0.03	0.248
TC	−0.88	**0.010**	0.13	0.500	−37.76	**0.031**	−4.36	0.469
LDL‐C	−0.15	0.678	−0.00	0.991	−28.81	**0.013**	−0.54	0.306
HDL‐C	−3.44	**0.010**	0.72	0.467	−0.31	0.028	0.42	0.842
Apo‐A1	−10.94	0.787	1.44	0.779	0.07	0.751	−0.01	0.554
Apo‐B	−16.51	0.538	−1.59	0.769	−9.03	0.256	0.02	0.481

*Note:* Bold values are statistically significant [*p* < 0.05].

Abbreviations: Apo‐A1, apolipoprotein A1; Apo‐B, apolipoprotein B; HDL‐C, high‐density lipoprotein cholesterol; LDL‐C, low‐density lipoprotein cholesterol; TC, total cholesterol; TG, triglycerides.

### 3.10 GRADE Assessment of Evidence Certainty

The overall quality of evidence was rated using the GRADE framework. Moderate certainty was assigned to findings related to TG and LDL‐C. The certainty for outcomes related to TC and Apo‐A1 was upgraded to very high, given the robustness and consistency of the data. Conversely, evidence regarding Apo‐B and HDL‐C was downgraded to low due to inconsistent and imprecise findings across studies (Tables 5 and 6).

**TABLE 6:** GRADE profile for the effect of walnut consumption on lipid profile and apolipoproteins in adults.

Outcomes	Risk of bias	Inconsistency	Indirectness	Imprecision	Publication bias	Quality of evidence
TG	No serious limitation	Very serious limitation^a^	No serious limitation	No serious limitation	No serious limitation	⊕ ⊕ ◯◯ Moderate
TC	No serious limitation	Serious limitation^a^	No serious limitation	No serious limitation	No serious limitation	⊕ ⊕ ⊕◯ High
LDL‐C	No serious limitation	Very serious limitation^b^	No serious limitation	No serious limitation	No serious limitation	⊕ ⊕ ◯◯ Moderate
HDL‐C	No serious limitation	Very serious limitation^b^	No serious limitation	Serious limitation^c^	No serious limitation	⊕◯◯◯ Low
Apo‐A1	No serious limitation	No serious limitation	No serious limitation	Serious limitation^c^	No serious limitation	⊕ ⊕ ⊕◯ High
Apo‐B	No serious limitation	Very serious limitation^b^	No serious limitation	Serious limitation^c^	No serious limitation	⊕◯◯◯ Low

Abbreviations: Apo‐A1, apolipoprotein A1; Apo‐B, apolipoprotein B; HDL‐C, high‐density lipoprotein cholesterol; LDL‐C, low‐density lipoprotein cholesterol; TC, total cholesterol; TG, triglycerides.

^a^ There is a significantly very high level of heterogeneity (*I* ^2^ > 75).

^b^ There is a significantly high level of heterogeneity (*I* ^2^ > 40).

^c^ There is no evidence of significant effects of walnut consumption.

### 3.11 Sensitivity Analyses

The leave‐one‐out sensitivity analyses showed that excluding any single study did not materially alter the pooled effect estimates for TG, TC, LDL‐C, HDL‐C, Apo‐A1, or Apo‐B. Across all outcomes, effect directions and statistical significance remained stable, indicating that no individual trial disproportionately contributed to the overall results. These findings confirm the robustness and reliability of the meta‐analytic estimates.

## 4 Discussion

This systematic review and dose–response meta‐analysis of walnut consumption provides the most comprehensive assessment to date of walnuts' impact on blood lipids and apolipoproteins. The findings confirm and extend prior evidence, showing that walnut‐enriched diets modestly but significantly improve the lipid profile of adults. In our analysis, walnut intake significantly reduced TC and LDL‐C concentrations compared with various control diets, without appreciably affecting HDL‐C levels. Crucially, our meta‐analysis is among the first to quantify walnuts' impact on apolipoproteins. We did not observe a significant overall change in Apo‐A1 and Apo‐B with walnut intervention.

These findings are consistent with prior large‐scale observational research demonstrating an association between walnut consumption and reduced cardiovascular risk. For instance, the Coronary Artery Risk Development in Young Adults (CARDIA) study, which followed 3092 individuals over 30 years, reported that higher long‐term walnut intake was associated with more favorable cardiovascular risk profiles (Yi et al. 2022). Specifically, individuals who regularly consumed walnuts demonstrated significant improvements in several cardiometabolic markers, including lower BMI, reduced waist circumference, decreased blood pressure, and reduced TG concentrations. Similarly, the PREDIMED (PREvención con DIeta MEDiterránea) trial showed a lower incidence of cardiovascular events among high‐risk individuals adhering to a Mediterranean diet that included walnuts (Guasch‐Ferré et al. 2021).

Several mechanistic factors help explain why and how walnuts produce the observed lipid changes. A key driver is the fatty acid composition of walnuts: they are high in polyunsaturated fats (especially ALA, ~2.5/30 g) and low in saturated fat. Replacing saturated fat in the diet with ALA‐rich walnuts leads to decreased hepatic cholesterol synthesis and upregulation of LDL‐C receptors, thereby enhancing clearance of LDL‐C particles from the bloodstream (Gao et al. 2022). This mechanism is analogous to the known cholesterol‐lowering effect of polyunsaturated fats in general, and our findings affirm that walnuts—being rich in ALA—follow this paradigm. Additionally, walnuts contribute bioactive compounds that further support lipid‐lowering. Each serving of walnuts provides a few grams of dietary fiber and a notable amount of phytosterols (plant sterols) (Nguyen and Vu 2023). These components can interfere with cholesterol absorption in the gut and promote the excretion of cholesterol and bile acids, thereby reducing plasma cholesterol levels over time (Holscher et al. 2018). The fiber content may also aid modest weight regulation by enhancing satiety, although in the trials we analyzed, caloric intake was often adjusted to prevent weight changes (Rock et al. 2017). Another emerging mechanism is the impact of walnuts on the composition of the gut microbiota (Holscher et al. 2018). Recent studies suggest that walnut consumption can enrich gut bacteria that produce secondary bile acids and short‐chain fatty acids, which in turn may influence lipid metabolism by increasing bile acid excretion and modulating hepatic cholesterol handling (Zeng et al. 2019). Although these microbiome‐mediated effects are still being elucidated, they provide an intriguing link between walnut intake and metabolic health. Furthermore, the rich antioxidant and polyphenol content of walnuts offers cardiometabolic benefits that complement lipid‐lowering (Sánchez‐González et al. 2017). Walnut‐derived polyphenols and L‐arginine have been shown in human studies to improve endothelial function and reduce oxidative stress, thereby stabilizing the vascular environment and preventing LDL‐C oxidation (McKay et al. 2010). Finally, walnuts may exert anti‐inflammatory effects by inhibiting the nuclear factor kappa B (NF‐κB) signaling pathway (Dai et al. 2024). Downregulation of NF‐κB activity may represent a potential mechanistic link between walnut consumption and improved health outcomes (Tan et al. 2022; Ghasemzadeh Rahbardar et al. 2025).

Based on our analyses, walnut consumption led to consistent reductions in TC and LDL‐C across all subgroups; however, its effects on TG varied, showing significance only in specific subpopulations. Several mechanisms may explain why TG reduction was observed in individuals with baseline TG ≥ 150 mg/dL but not in those with normal TG levels. Patients with elevated TG typically exhibit greater hepatic VLDL overproduction and impaired clearance; thus, interventions that improve lipid metabolism, such as increased intake of PUFAs, α‐linolenic acid, and fiber from walnuts, tend to produce larger absolute reductions when baseline dyslipidemia is present. This “greater room for improvement” phenomenon is well‐documented in dietary lipid‐lowering trials (Rerup et al. 2021).

Short‐term trials (< 12 weeks) also demonstrated significant reductions in TG, whereas longer interventions did not. This may reflect early metabolic responsiveness to PUFA‐rich foods, including rapid reductions in hepatic VLDL synthesis and enhanced lipoprotein lipase activity (Coiffier et al. 1987), which tend to plateau over time as metabolic adaptation occurs. Several dietary intervention studies have likewise demonstrated that TG responses are more favorable in acute or short‐term interventions (Karimi, Javadi, Sharifi, et al. 2025; Karimi, Karimi, et al. 2025; Mohammadi et al. 2025) diminishing over prolonged follow‐up periods as weight, dietary adherence, and energy‐compensation behaviors stabilize.

The TG‐lowering effect was also significant only among participants with obesity (BMI ≥ 30 kg/m^2^). Obese individuals typically exhibit insulin resistance, hepatic steatosis, and higher baseline TG levels, all of which increase responsiveness to interventions targeting hepatic lipid export (Liu et al. 2021). Walnuts, via their PUFA profile, antioxidant content, and anti‐inflammatory properties, may have a disproportionately greater impact in improving hepatic lipid handling in this metabolic phenotype (González‐Périz et al. 2009), whereas individuals with normal BMI or mild overweight may show minimal improvement due to relatively normal baseline TG physiology.

In our meta‐analysis, walnut consumption resulted in statistically significant reductions in TG (−6.24 mg/dL), TC (−6.39 mg/dL), and LDL‐C (−5.68 mg/dL). To determine the clinical relevance of these effects, it is essential to compare them with the minimal clinically important difference (MCID). Recent evidence suggests MCID thresholds of approximately 0.26 mmol/L (≈10 mg/dL) for TC, 0.10 mmol/L (≈3.9 mg/dL) for LDL‐C, and 0.09 mmol/L (≈8.0 mg/dL) for TG (Goldenberg et al. 2021; Jibril et al. 2022). Based on these cut‐offs, the reductions observed in TG and TC in the overall sample did not reach the MCID threshold, indicating limited clinical relevance despite statistical significance. The reduction in LDL‐C slightly exceeded the MCID, suggesting a potentially meaningful clinical effect. Given that the TG reduction in our analysis was substantially below this range, its impact on cardiovascular risk is likely minimal. However, subgroup analyses indicate that, among individuals with dyslipidemia, who typically have higher baseline lipid levels, the lipid‐lowering effects of walnuts were greater and, in several cases, exceeded MCID thresholds for TG, TC, and LDL‐C. Thus, while the clinical impact in the general population may be modest, walnut intake may offer clinically meaningful improvements in lipid levels among individuals with dyslipidemia.

Although subgroup analyses indicated that walnut intake below 50 g/day was associated with a statistically significant increase in HDL‐C concentrations, the practical feasibility and clinical relevance of this finding warrant careful consideration. Even at this high intake level, the observed increase in HDL‐C was modest, approximately 1.1 mg/dL. Given that the MCID for HDL‐C is estimated at 0.10 mmol/L (≈3.9 mg/dL), the magnitude of change associated with elevated walnut consumption does not meet the threshold for clinical significance. Consequently, recommending walnut intakes specifically to improve HDL‐C levels is not supported by clinically meaningful evidence.

This meta‐analysis possesses several notable strengths that enhance the credibility of its findings. Our study provides the most comprehensive evaluation to date of walnut consumption and its effects on lipid profiles and apolipoproteins, drawing from a wide range of RCTs across diverse populations and settings. Key strengths include the inclusion of recent long‐term trials, application of dose–response modeling to assess walnut intake quantitatively, and the specific analysis of Apo‐A1 and Apo‐B, markers often overlooked in prior reviews. Despite these strengths, notable heterogeneity across studies and limited data on apolipoproteins, particularly Apo‐A1, may affect the precision and generalizability of the findings. Nonetheless, the results offer valuable insights into the cardiometabolic benefits of walnuts. Moreover, the study protocol was not prospectively registered in PROSPERO or any other public registry. A further limitation relates to the background diets of participants in the included studies. Most trials did not report detailed information on habitual dietary intake, and control diets were generally described only as “habitual diet” without specification of macronutrient composition or adherence to dietary patterns such as Mediterranean or low‐fat diets. As dietary context may influence lipid responses to walnut intake, the lack of detailed reporting may limit the generalizability of our findings.

## 5 Conclusion

This systematic review and meta‐analysis provide evidence that walnut consumption favorably influences blood lipid profiles in adults, particularly by reducing TC, LDL‐C, and TG. In contrast, no significant effects were observed for HDL‐C, Apo‐A1, or Apo‐B. These results align with, yet extend, previous findings by reinforcing walnuts' lipid‐lowering potential and highlighting their role as a practical dietary intervention. Taken together, the evidence suggests that incorporating even moderate amounts of walnuts—such as a few handfuls per day—into the habitual diet may contribute to clinically relevant improvements in lipid‐related cardiovascular risk markers and overall cardiometabolic health.

## Author Contributions

All authors confirm that the authorship list is final, that they have made substantial contributions to the study, and that they take responsibility for the integrity of the work. Ghazal Mashayekhi, Arvin Porkar Rezaeyeh, and Zahra Shouhani performed the literature screening and data extraction. Omid Asbaghi conducted the statistical analyses. Damoon Ashtary‐Larky prepared and supervised the original draft. Mehdi Karimi contributed to the study conceptualization and critically reviewed and revised the manuscript. Ali Hosseini and Moslem Naderian provided overall supervision. All authors critically reviewed and approved the final manuscript.

## Funding

This work did not receive any specific grant from funding agencies in the public, commercial, or not‐for‐profit sectors.

## Ethics Statement

The authors have nothing to report.

## Consent

The authors have nothing to report.

## Conflicts of Interest

The authors declare no conflicts of interest.

## Data Availability

All data used in this meta‐analysis were extracted from published studies. The datasets supporting the findings of this study are available from the sources cited in the manuscript. Additional information on the data that support the findings of this study is available from the corresponding author upon reasonable request.
